# Early Loading of Mandibular Molar Single Implants: 1 Year Results of a Randomized Controlled Clinical Trial

**DOI:** 10.3390/ma13183912

**Published:** 2020-09-04

**Authors:** Jungwon Lee, Young-Jun Lim, Bongju Kim, Ki-Tae Koo

**Affiliations:** 1 Department of Periodontology, One-Stop Specialty Center, Seoul National University Dental Hospital, Seoul 03080, Korea; jungwonlee.snudh@gmail.com; 2 Department of Prosthodontics and Dental Research Institute, School of Dentistry, Seoul National University, Seoul 03080, Korea; 3 Dental Life Science Research Institute & Clinical Translational Research Center for Dental Science, Seoul National University Dental Hospital, Seoul 03080, Korea; bjkim016@gmail.com; 4 Department of Periodontology and Dental Research Institute, School of Dentistry, Seoul National University, Seoul 03080, Korea; periokoo@snu.ac.kr

**Keywords:** dental implant, early loading, primary stability, ISQ value, stability dip

## Abstract

The purpose of this study was to compare the implant survival, peri-implant marginal bone level, and peri-implant soft tissue of three different types of implants. This was performed with an early loading protocol, using a complete digital workflow, for one year of follow-up. Twenty-four patients with a single missing tooth in the mandibular posterior region were randomly assigned to the control group (SLActive Bone level implant; Institut Straumann AG, Basel, Switzerland), experiment group 1 (CMI IS-III Active implant; Neobiotech Co., Seoul, Korea), and experiment group 2 (CMI IS-III HActive implant; Neobiotech Co., Seoul, Korea). For each patient, a single implant was installed using the surgical template, and all prostheses were fabricated using a computer-aided design/computer-aided manufacturing system on a 3-dimensional model. A provisional prosthesis was implanted at 4 weeks, and a definitive monolithic zirconia prosthesis was substituted 12 weeks following the implant placement. The implant stability quotient (ISQ) and peri-implant soft tissue parameters were measured, and periapical radiographs were taken at 1, 3, 4, 8, 12, 24, 36, and 48 weeks after implant placements. Seven implants in the control group, nine implants in the experiment 1 group, and eight implants in the experiment 2 group were analyzed. There were no significant differences among the three groups in terms of insertion torque, ISQ values between surgery and 8 weeks of follow-up, marginal bone loss at 48 weeks of follow-up, and peri-implant soft tissue parameters (*P* > 0.05). Statistically significant differences in ISQ values were observed between the control and experiment 1 groups, and the control and experiment 2 groups at the 12 to 48 weeks’ follow-ups. Within the limits of this prospective study, an early loading protocol can be applied as a predictable treatment modality in posterior mandibular single missing restorations, achieving proper primary stability.

## 1. Introduction

According to Branemark’s original protocol, in order to obtain a direct bone-to-implant interface (osseointegration), dental implants need to be submerged under the soft tissue and left for 3–6 months [1]. The rationale of the unloading period is to avoid movements of the dental implant during the healing process of the bone that may interfere with osseointegration between the bone and implant, resulting in fibrous encapsulation [2].

Many efforts have been made to shorten the restoration period, in order to reduce patient discomfort. First, several studies have shown that submerging the dental implants under the soft tissue is not necessary for successful implant restoration [3,4,5,6]. Second, implant surfaces have been improved to reduce treatment time including an early loading protocol following implant placement [7]. From this evidence, implant placement can be performed following an early loading protocol with non-submerging, in order to reduce the restoration periods.

Recent advances in 3-dimensional (3D) imaging and computer-aided design (CAD)/computer-aided manufacturing (CAM) have enabled accurate diagnosis and created a surgical guide for implant placement [8]. A virtual model can be created by coordinating radiographic data (Digital Imaging and Communications in Medicine; DICOM) using cone beam computed tomography (CBCT) images and standard tessellation language (STL) files from intraoral scanning using digital software. Detailed surgical planning can increase predictability in surgery at the proper location of the implant by reducing spontaneous intraoperative decisions or deviations from the surgical procedure [9,10].

Implant manufacturing technologies have been improved to enhance implant stability. In terms of macrodesign, a tapered implant can be used to exercise compression of the surrounding bone during the implant insertion protocol, increasing implant primary stability [11]. In addition, the reduced diameter of the apical part of the implant means it can be accommodated in the available alveolar bone, thereby preventing bone perforation [12,13].

Implant surface modification has been reported to influence primary and secondary implant stability. A study by Salermo et al. showed that modified implant surfaces are well preserved even after implant placement [14]. The area of the bone-to-implant interface can be extended by increasing the implant surface roughness, resulting in an improvement in the primary stability of the implant [15,16]. In addition, implants with increased surface roughness promote osseointegration between the implant and bone, thus reducing the waiting period for bone healing before loading [17,18]. Recently, efforts have been made to minimize the time when stability dips occur by increasing hydrophilicity or bioactivity of the implant surface to promote blood clot formation around the implant and growth factors to move around the implant [19,20].

The purpose of this study was to compare the implant survival, peri-implant marginal bone level, and peri-implant soft tissue of three different types of implants with an early loading protocol using a complete digital workflow with a one-year follow-up.

## 2. Materials and Methods

### 2.1. Study Ddesign

All procedures were conducted according to the Declaration of Helsinki on experimentation involving human subjects [21]. This clinical trial was approved by the Institutional Review Board of the Seoul National University Dental Hospital (IRB No. CDE18001). Informed consent about the nature of the study was obtained from all participants. The manuscript was prepared according to the Consolidated Standards of Reporting Trials (CONSORT) guidelines [22].

The subjects were patients with single missing mandibular posterior teeth at least 3 months after extraction. A surgical guide was prepared using digital impressions with an intraoral scanner (Trios3; 3Shape, Copenhagen, Denmark) and CBCT data before surgery. Dental implant type was allocated randomly before surgery with a computerized random number using software (Excel 16.0, Microsoft, Redmond, Washington, WA, USA). A provisional restoration was installed according to the early loading protocol, and the final prosthesis was mounted 12 weeks (3 months) after implant placement. Implant success rates and implant stability were evaluated and compared between the three groups for 13 months.

The study design was a three-arm randomized controlled trial. Three different types of implants were used in this study, as follows: SLActive bone level implant (Institut Straumann AG, Basel, Switzerland; surface roughness (Ra) = approximately 1.8 μm [23]; contact angle = 0° [24]) in the control group, CMI IS-III Active implant (Neobiotech Co., Seoul, Korea; Ra = approximately 3.5 μm; contact angle = 109.2° [20]) in the experimental group 1, and CMI IS-III HActive implant (Neobiotech Co., Seoul, Korea; Ra = approximately 3.5 μm; contact angle = approximately 4° [20]) in experiment group 2.

**Figure 1:**
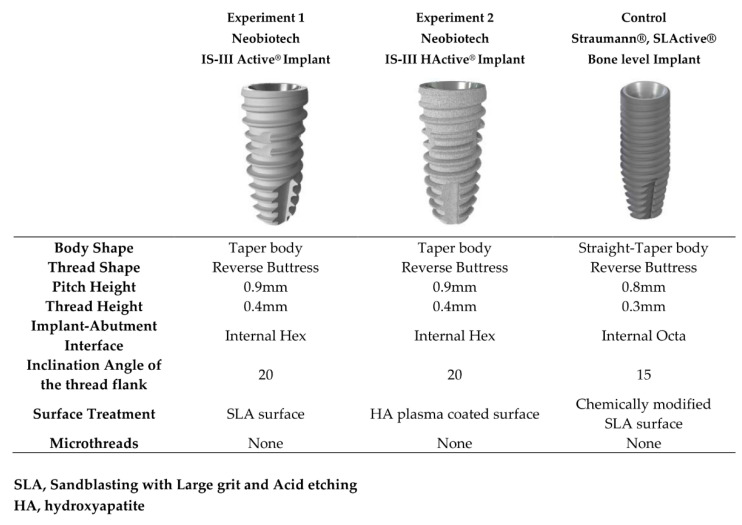
Characteristics of the implant systems used in this study: CMI IS-III active (Neobiotech, Seoul, Republic of Korea), CMI IS-III Hactive (Neobiotech, Seoul, Korea), and SLActive bone level (Straumann, Basel, Switzerland).

The characteristics of the implant types used in this study are shown in Figure 1.

### 2.2. Sample Size Calculation

The sample size was calculated based on a previous study related to early loading with chemically modified surface implants [25]. The total number of patients was calculated using sample size calculation software G*power 3.1.9.2 (University of Düsseldorf, Düsseldorf, Germany) using the analysis of variance (fixed effects, omnibus, one-way) of parallel design. The random allocation ratio among groups (λ) was 1:1:1 (control group: experiment group 1: experiment group 2). The expected probability of survival of the control, experiment 1, and experiment 2 groups were 98%, 96%, and 97%, respectively, and the standard deviation within each group was 0.01. The alpha error probability was set to 0.05, and the statistical power was 0.85. This indicated that seven subjects were needed in each group, and so nine subjects were included in each group considering an estimated dropout rate of 25%. Therefore, the total number of subjects required in this study was 27.

### 2.3. Participants

This study included 27 subjects at a single institute in Seoul National University Dental Hospital, Seoul, Republic of Korea. A total of 102 potential participants were recruited using subway advertisements.

The following inclusion criteria were applied: (a) 18 years of age or older; (b) a single tooth missing in the posterior mandibular region, with at least 3 months having passed after the tooth extraction; (c) the ability to undergo implant surgery and restoration; (d) sufficient bone volume in the site to accommodate implant placement without any need for bone augmentation (the residual bone height between the alveolar crest and the inferior alveolar nerve had to be more than 12 mm, the buccolingual width had to be greater than 7 mm, and the mesiodistal length was 6–8 mm in the premolar region; the residual bone height between the alveolar crest and the inferior alveolar nerve had to be more than 12 mm, the buccolingual width had to be greater than 8 mm, and the mesiodistal length had to be 12 mm in the molar region); and (e) normal occlusal plane of the opposite teeth and no missing areas in the opposite jaw. The exclusion criteria were (a) pregnancy, (b) myocardial infarction within 1 year, (c) bleeding disorders or need for blood anticoagulants for surgery, (d) any systemic disease affecting the surgery or restoration procedure; (e) mental illness; (f) allergy to implant materials; (g) adjacent periodontally compromised teeth; (h) parafunctional habits or disorders; (i) lack of interocclusal space; (j) bone quality type D4; (h) insertion torque of less than 35 Ncm or greater than 45 Ncm; and (i) implant stability quotient (ISQ) value <65.

### 2.4. Treatment Procedure

#### 2.4.1. Preoperative Diagnosis and Preparation

**Figure 2:**
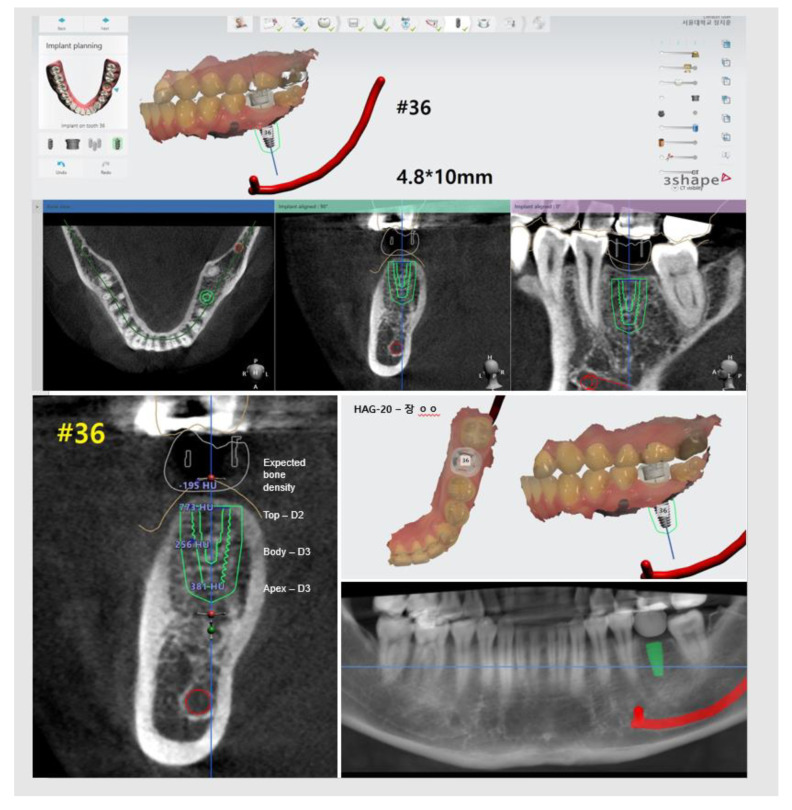
Patient-specific analytical procedures including the virtual implant position and bone density; these were performed using Implant Studio software for the preparation of a surgical guide.

All subjects underwent preoperative diagnosis and preparation for implant surgery and prosthetic restoration planning. A panoramic X-ray and CBCT (CS9300, Carestream Health, Rochester, NY, USA) were used for diagnosis, and a digital impression was taken using an oral scanner (Trios3; 3Shape, Copenhagen, Denmark). Based on CBCT images and digital impression data, implant placement and prosthetic restoration were planned using Implant Studio software (3Shape, Copenhagen, Denmark), taking into account the anatomical structure and intermaxillary relationship (Figure 2).

According to this plan, surgical templates, customized titanium abutments, and provisional prostheses were produced.

#### 2.4.2. Implant Placement

**Figure 3:**
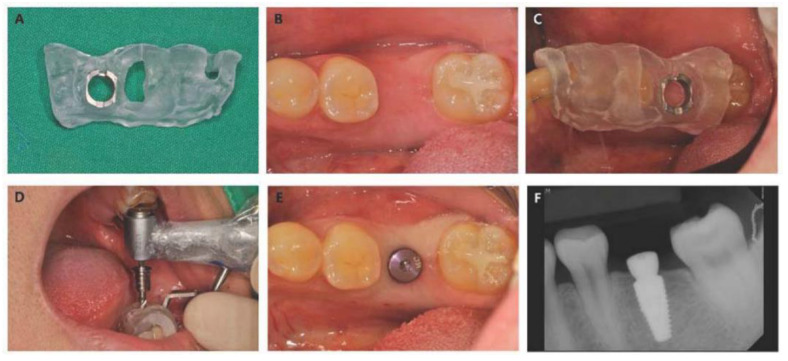
Representative photograph and radiograph of the implant surgery. (**A**) Clinical photograph of the surgical template, (**B**) clinical photograph before surgery, (**C**) placement of surgical template, (**D**) drilling procedure of flapless surgery, and (**E**) clinical photograph after implant placement. (**F**) Periapical radiograph after implant placement.

Implant placement was performed by two experienced periodontists using a surgical template (Figure 3).

If the buccolingual width of the attached gingiva was 8 mm or more, the implant was placed following a punch technique without incision. If the buccolingual width of the attached gingiva was less than 8 mm, the implant was placed with minimal incision. The drilling procedure was performed in accordance with the manufacturer’s recommendation protocol for early loading. In the control group, the bone quality was divided into type 1, large homogenous cortical bone; type 2, thick cortical layer surrounding a dense medullar bone; type 3, thin cortical layer surrounding a dense medullar bone; and type 4, thin cortical layer surrounding a sparse medullary bone, when osteotomy was performed with a 2.2-mm straight drill [26]. In contrast, in the case of experiment group 1 or 2, the bone quality was recorded according to the modified Misch’s classification as in a previous study [27]. In brief, the bone was divided into 3 parts depending on the depth, and the bone density was divided into D1, D2, and D3. In all three groups, the poor bone (type 4 or D4) subjects were excluded in this study.

The surgical template was removed after the implant was placed at the planned location and depth in order to measure the insertion torque. A small amount of bleeding was observed in the surgical site (Figure 3E). The target values of implant insertion torque and ISQ value measured with Osstell Mentor (Integration Diagnostics AB, Göteborg, Sweden) were 35–45 N·cm and greater than 65 N·cm, respectively. If the insertion torque was out of the range of 35 to 45 N·cm, or the ISQ value was less than 65, the subject was excluded from the experiment.

#### 2.4.3. Prosthesis Procedure

ISQ values measured on the day of surgery and 1 week, 3 weeks, and 4 weeks after surgery were greater than 65; pre-fabricated customized abutment and provisional prosthesis were inserted 4 weeks after the surgery. The prepared titanium customized abutment was secured to the implant with 20 N·cm, and a provisional prosthesis was delivered with temporary cement. In the case of excursion, the articulating paper should not be in contact, and the occlusion was adjusted so that the bite force was applied to the long axis of the implant. After the restoration was installed, a periapical view radiograph was taken using a digital sensor holder (EEZEE-Grip, Dentsply Rinn, York, PA, USA).

The final prosthetic procedure was performed using a digital prosthetic workflow 8 weeks after implant placement. Digital impression was obtained with a pre-abraded titanium customized abutment with an oral scanner (Trios3; 3Shape, Copenhagen, Denmark). The monolithic zirconia final crown of a definitive fixed screw and cement-retained prosthesis (SCRP) was inserted 12 weeks following implant surgery. The screw hole within the prosthesis acted as a vent hole for cement for the escape of excess luting cement. The residual cement in subgingival margin was checked with explorer. After prosthesis cementation, the hole was sealed with Teflon tape and resin.

#### 2.4.4. Measurement of Implant Stability

The insertion torque value that measured in the process of implant installation during surgery was recorded as peak insertion torque. Implant stability was measured using an Osstell Mentor at the following times: at implant placement, 1, and 3 weeks after implant placement; again at 4 weeks (provisional prosthesis insertion), 8 weeks, and 12 weeks (final prosthesis insertion); and also at 24, 36, and 48 weeks after implant placement. The ISQ value was measured on the mesial, distal, buccal, and lingual sides and represented as an average.

#### 2.4.5. Measurement of Marginal Bone Loss

**Figure 4:**
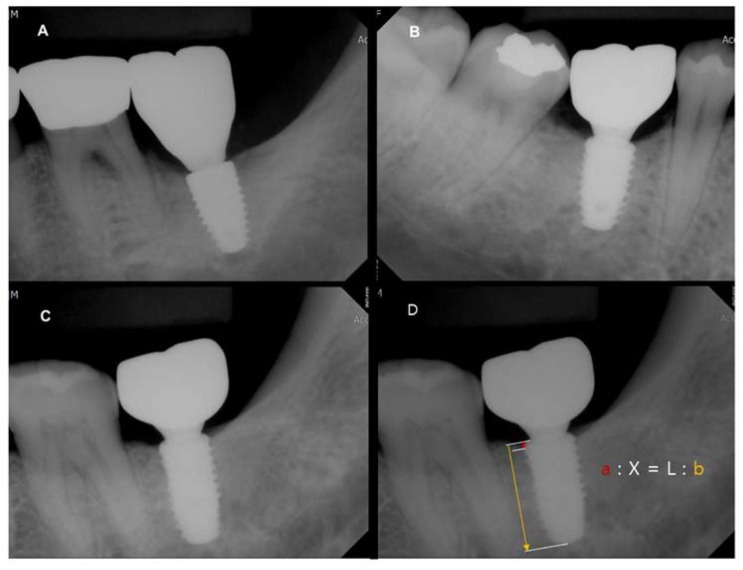
Comparison of marginal bone loss 24 weeks after surgery. (**A**): control group; (**B**): experiment 1; (**C**): experiment 2; (**D**): measurement of marginal bone loss (MBL) on periapical radiograph (X: distance between platform and peri-implant bone crest).

Periapical radiographs were taken to evaluate the amount of marginal bone changes following the implant surgery and 48 weeks after implant placement. The ratio between the actual distance (L) and the distance on the radiograph (b) was calculated. In the radiographs taken at implant placement and 48 weeks after implant placement, the distance between the alveolar crest and implant platform (a) was measured on the radiograph and converted to the actual distance (X) between the alveolar crest and implant platform by applying the ratio (Figure 4).

The marginal bone changes between the implant placement and 48 after the implant placement were measured at each mesial and distal area, and then the average value was calculated, Equation (1):

(1) $$X=\frac{b}{L}\times a$$

#### 2.4.6. Recall Visit Procedures and Implant Evaluation

All patients were scheduled for recall visits at 24, 36, and 48 weeks after implant placement. Clinical and radiographic evaluations were conducted during the follow-up period. Full mouth occlusion was investigated, and the ISQ values were assessed at every appointment. In addition, soft tissue evaluation including plaque index, calculus index, sulcus bleeding index, and widths of keratinized mucosa were examined, and periapical radiographs were taken. Any complications including clinically detectable mobility, pain or other symptoms of discomfort or ongoing pathologic processes, peri-implantitis with suppuration, or continuous radiolucency around the implant was evaluated during the follow-up. 

### 2.5. Statistical Analysis

Statistical analysis comparing the three groups was performed based on the intention to ireat (ITT) and the per protocol (PP) analyses. The χ2 test for categorical variables and the Kruskal–Wallis test for differences between groups were used (SigmaPlot 14.0, Systat Software Inc., San Jose, CA, USA). Pairwise comparisons were performed using the Mann–Whitney U test in the cases with significant differences according to the Kruskal–Wallis test. The level of significance (*P* = 0.05) was adjusted according to the Bonferroni correction method. Two-way repeated measures analyses of variance were performed after the verification of sphericity using the Huynh–Feldt method to evaluate the differences in the patterns of ISQ changes over time.

## 3. Results

### 3.1. Participants and Implants

**Figure 5:**
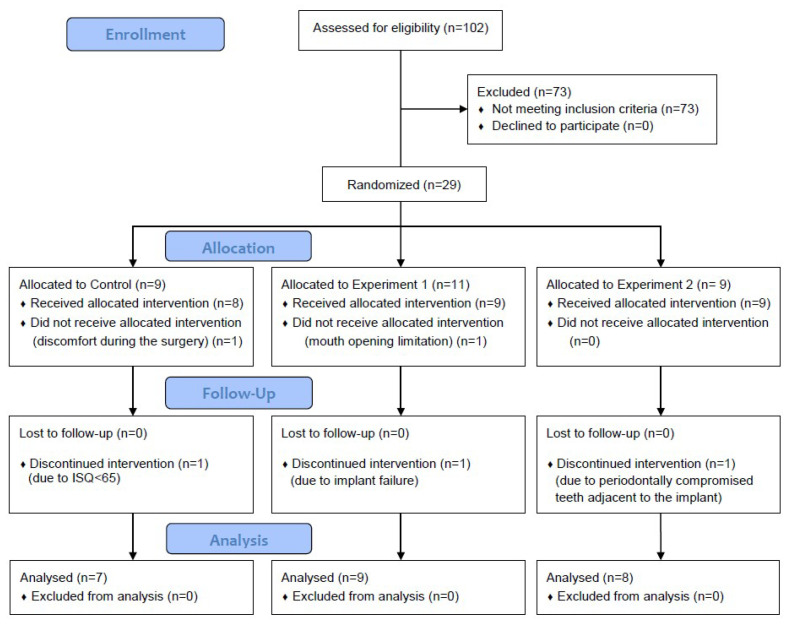
Flow diagram of this study.

A total of 102 candidates were recruited using subway advertisements. A total of 73 subjects were excluded during the screening process, and 29 patients were finally selected for the study. Two subjects were excluded during the surgery because they were not suitable for conducting the clinical trial due to mouth limitations or patient discomfort caused by proximity to the nerve. One patient was excluded due to adjacent periodontally compromised teeth identified during follow-up. One patient was excluded due to low implant stability values (ISQ < 65), and one patient was excluded due to implant failure during follow-up. As a result, the data from 24 implants in 24 participants were analyzed for the present study (Figure 5).

The mean age of the 24 subjects was 50.3 ± 10.55 years, ranging from 26 to 65 years. They participated in this study from January 2018 to October 2019 (Table 1).

Seven implants were placed in the first mandibular molars, fifteen implants were placed in the second mandibular molars, and two implants were placed in the second mandibular premolars. Seven implants were performed in the control group, nine in the experiment 1 group, and eight in experiment 2 group.

### 3.2. Comparison of Implant Stability among the Three Different Types of Implants

**Figure 6:**
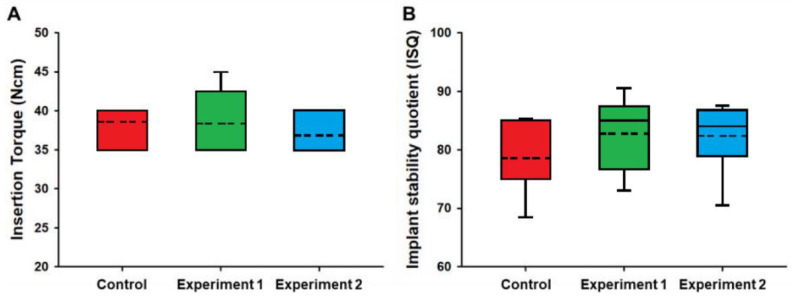
Box graph of primary stability between control, experiment 1, and experiment 2. (**A**): insertion torque (*P* = 0.519, Kruskal–Wallis test); (**B**): implant stability quotient (ISQ) (*P* = 0.391, Kruskal–Wallis test).

The primary stability was investigated using the peak insertion torque and ISQ value at the time of implant placement (Figure 6).

In this clinical study, the insertion torque values and ISQ values in all patients met the inclusion criteria. The insertion torque values were similar in the three groups (*P* = 0.559). The ISQ value was lower in the control group than in experiment group 1 or 2, but there were no statistical differences (*P* = 0.389).

**Figure 7:**
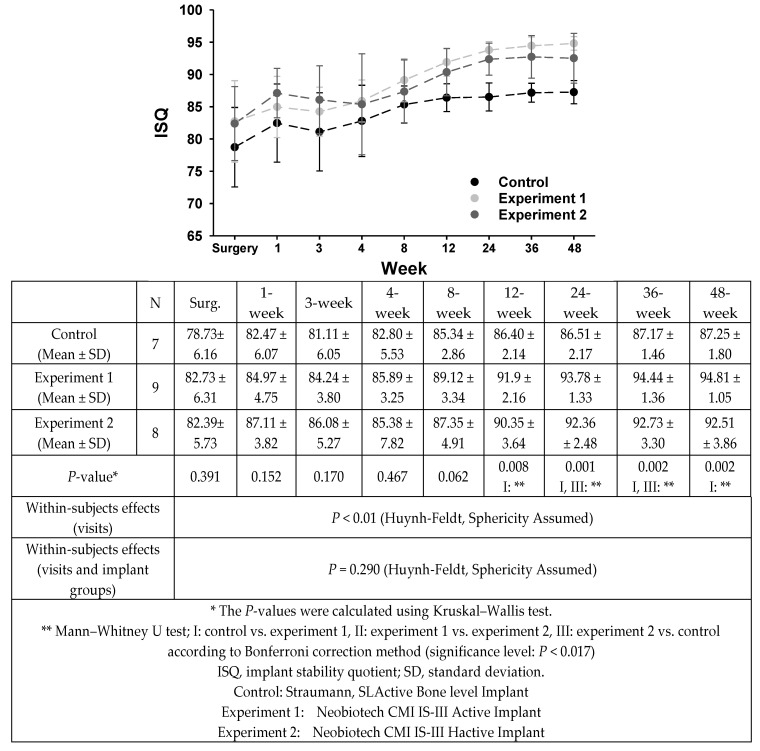
Comparison of stability in terms of the pattern of changes in implant stability quotients (ISQ) during the 48-week observation period after implant surgery.

The measurements of the ISQ values in the three groups are presented in Figure 7.

The ISQ values progressively increased in all control and experimental groups, which showed a remarkable increase 4 weeks after the surgery. The ISQ values of experiment groups 1 and 2 were relatively higher than those of the control group at the same observation times. In particular, ISQ had the highest value in the experiment 2 group (CMI IS-III HActive) until 4 weeks after surgery. There were no statistical differences in all three groups at 8 weeks (*P*-value > 0.05).

Overall, the ISQ values progressively increased in all control and experimental groups. There was a period in which the increase in ISQ was stagnant between 2 and 4 weeks after the surgery; however, it increased again after 4 weeks. The ISQ values of experimental groups 1 and 2 were relatively higher than those of the control group at the same observation time. In particular, ISQ had the highest value in the experiment 2 group (CMI IS-III HActive) until 4 weeks after the surgery, and experimental group 1 (CMI IS-III Active) showed the highest value 8 weeks after the surgery. There was no statistical difference in any of the three groups until 8 weeks after the surgery. ISQ values 12, 24, 36, and 48 weeks after surgery were significantly higher in the experiment group 1 and 2 groups than in the control group (*P* < 0.05).

### 3.3. Comparison of Marginal Bone Loss among Three Different Types of Implants

The marginal bone changes showed little bone resorption at the 12-, 24-, and 48-week follow-ups, nor 24 weeks after implant placement in the control group and experiment groups 1 and 2. The amount of bone resorption was found to be relatively low in experiment group 1 compared to the control group or experiment group 2, but there were no statistical differences in all groups (Table 2, *P* > 0.05).

### 3.4. Evaluation of the Peri-implant Soft Tissue Parameters and Success Rates of Three Types of Implants

**Figure 8:**
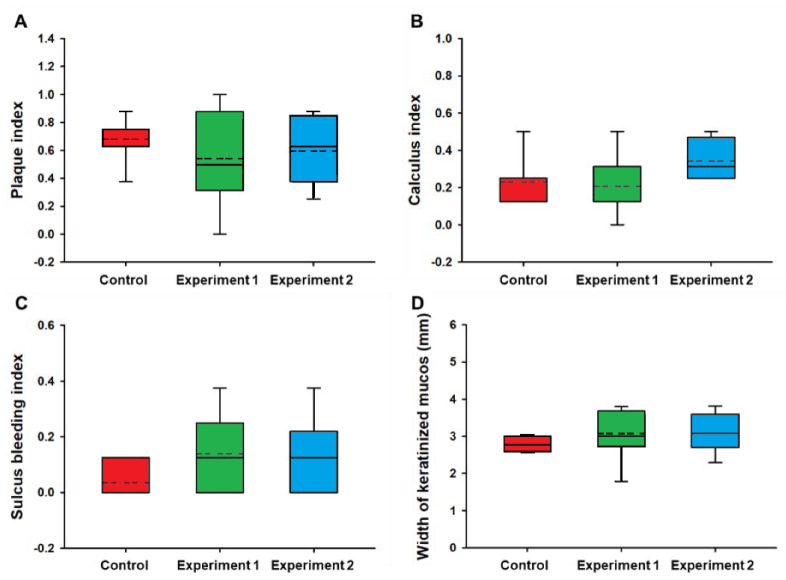
Box graph of peri-implant soft tissue parameters between the control, experiment 1, and experiment 2 groups at the 48-week follow-up. (**A**) Plaque index: score 0, no detection of plaque; score 1, plaque only recognized by running a probe across the smooth marginal surface of the implant; score 2, plaque can be seen by the naked eye; score 3, abundance of soft matter. (**B**) Calculus index: score 0, no detection of calculus; score 1, supragingival calculus covering ≤ 1/3 exposed tooth surface; score 2, supragingival calculus covering >1/3 but <2/3 tooth surface, flecks of subgingival calculus in cervical margin; score 3, supragingival calculus covering >2/3 surface, continuous band of subgingival calculus. (**C**) Sulcus bleeding index: score 0, no bleeding when a periodontal probe is passed along the gingival margin adjacent to the implant; score 1, isolated bleeding spot visible; score 2, blood forms a confluent red line on margin; score 3, heavy or profuse bleeding. (**D**) Width of keratinized mucosa (*P* = 0.668, 0.118, 0.079, 0.293, respectively, determined by the Kruskal–Wallis test).

All peri-implant evaluations including plaque index, calculus index, and sulcus bleeding index conducted at the 48-week follow-ups following implant placement demonstrated healthy peri-implant tissues, with no statistically significant differences among the three groups (Figure 8A–C).

Furthermore, there were no statistically significant differences in the width of the keratinized mucosa among the three different types of implant groups (Figure 8D). At the end of the 48 weeks of observation, all implants except one implant in the control group showed implant success according to the success criteria. 

## 4. Discussion

This randomized controlled clinical study compared implant survival, peri-implant marginal bone level, and peri-implant soft tissue of three different types of implants, with an early loading protocol, using a complete digital workflow. One implant in the control group failed and the others survived, with minimal changes in marginal bone level. At the end of the 48 weeks of observation, just one implant in the control group showed implant failure in accordance with the success criteria. All implants showed little change in the marginal bone level at the 48-week follow-up (0.28 ± 0.25 mm, 0.20 ± 0.15 mm, 0.34 ± 0.22 mm in the control, experiment 1, experiment 2 group, respectively). When considering the ranges of errors in evaluating periapical radiographs [28] and physiologic peri-implant bone changes after implant placement [29,30], the outcomes of this clinical study can be deciphered as an acceptable peri-implant marginal bone stability.

In this study, the inclusion criteria for early loading protocol was insertion torque values between 35 and 45 N·cm. Although adequate primary stability is required for predictable results of early loading protocol, the criteria of insertion torque value for immediate or early loading showed various ranges of values. A recent systematic review demonstrated that the criteria of insertion torque value for immediate or early loading was heterogeneous including ≥15 N·cm (12.5%), ≥20 N·cm (4.2%), ≥30 N·cm (20.8%), ≥35 N·cm (50%), ≥40 N·cm (8.3%), or ≥45 Ncm (4.2%) in the previous studies [31]. In this study, the inclusion criteria of insertion torque value was set the most adopted value in the previous studies in order to perform early loading more safely because confounding factors including poor bone quality, systemic condition, or age can jeopardize implant stability. 

Hydroxyapatite (HA) has been applied to implant surfaces to facilitate bone-to-implant contact in various studies [32,33]. Some studies on HA-coated implants showed a high survival rate; however, another study with long-term follow-up reported poor predictable survival rates, which was 77.8% at 8-year follow-ups [34]. It is known that most of these unpredictable results of HA-coated implants are accompanied by peri-implant marginal bone loss caused by the peeling phenomenon of the HA coating [35]. As the HA coating layer comes off the implant surface, it can act as a causative factor of the inflammatory reaction.

In order to solve these mechanical issues and make the most of the osteoconductive activity of HA, a technology related to thin HA coatings on the implant surfaces has been developed and implemented [36,37]. According to previous studies, the appropriate thickness for using bio-reactivity while reducing the mechanical problem of the HA coating is known to be a few micrometers. The bio-activity of the HA coating was confirmed to some extent by the highest ISQ value of the experiment 2 group before loading, and the safety of the HA coating was confirmed by stable marginal bone changes at the 1 year follow-up in this study (Figure 6).

Another type of surface treatment, a chemically modified sandblasting and large-grit acid etching (SLA) surface, is known to enhance hydrophilicity, facilitating bone apposition during the early stages of bone healing [19]. Chemically modified SLA surface implants showed a clinically acceptable marginal bone loss, with a high implant survival for early loading, although conventional SLA surface implants showed similar implant survival rates [38]. The present outcomes were in agreement with the previous study in that all three types of implants showed small marginal bone loss and a high implant survival rate for the 1-year follow-up period (Table 2).

Unlike preclinical studies of chemically modified SLA implants and HA-coated implants increasing osseointegration at the early bone healing stage [20], this clinical study failed to confirm the clinical benefits of HA-coated implants or chemically modified SLA implants compared to conventional SLA implants. The effect of implant surface modification might be masked by inclusion criteria such as sufficient bone quantity and good bone quality for this study. In clinical situations where the bone quantity is insufficient and the quality of bone is poor, it is considered that the effect of implant surface modification may be maximized, necessitating the use of those implants.

Overall, the ISQ values progressively increased in all types of implants, with a stagnant period between 2 and 4 weeks, and an increasing period between 4 and 24 weeks following implant placement. At 4 weeks after implant placement, implant prostheses were delivered. It seems that the ISQ values might increase after loading progressively to some extent. A previous human study demonstrated that loading might stimulate bone remodeling around the implant and increase lamellar bone formation [39]. Physiologic loading might increase bone remodeling and lamellar bone formation, resulting in increased ISQ values in this study.

The macro-design of the implant seems to affect the ISQ values post-loading. In this study, the experiment 1 and experiment 2 group showed higher ISQ values after 12 to 48 weeks of observation (post-loading period) compared to the control group. Some differences were observed in the macro-design between the experimental and control groups in terms of tapering, pitch height, thread height, and inclination angle of the thread flank. A previous study reported that 0.9 mm of the pitch height of the implant showed an improved stress distribution compared to 0.8 mm of the pitch height of the implant [40]. Interestingly, similar results were observed in the previous study showing no significant differences in ISQ values before loading between different macro-design implants, in contrast, showing significant differences in ISQ values after loading between different macro-design [41]. Implant macro-threads in the coronal part seems to distribute the occlusal stress evenly to the crestal bone, reducing stress concentration per unit area.

Accurate implant placement is important for esthetic and functional implant prosthesis. A surgical guide for implant placement was created with the 3D imaging and CAD/CAM technology. In addition, elaborate surgical planning can improve predictability in surgery at the proper location of the implant [9,10]. However, in order to fabricate a surgical guide, the patient’s radiographic data and a virtual model should be provided to the laboratory, and as a result, the overall time to manufacture surgical guide was about 7–10 days, making it difficult to apply in clinical use. In the future, there should be technological advances to fabricate surgical guides within an hour in the clinic in implant dentistry.

Various literatures have emphasized the importance of case selection in relation to early loading [25,38,41]. In this study, patients with proper implant stability, bone quality, healthy periodontal status adjacent the implant site, and no parafunction, were the inclusion criteria. For a successful early loading protocol, these systemic and local conditions should be fully considered and applied in clinical situation.

The limitation of this study is the small sample size and short follow-up period. As in previous studies, many technical and biological complications related to dental implants occur at least 3 years post-loading [42,43]. A systematic review demonstrated that longitudinal studies with long term follow-up exceeding 5 years should be evaluated to investigate implant success [44]. In addition, the number of sample size per each group might be small when considering the confounding factors including different bone quality, implant diameters, and implant length in the present study even with the sample size calculation. Therefore, subsequent randomized controlled trials with larger sample sizes and longer follow-up periods comparing with conventional loading are needed in order to secure this treatment modality.

## 5. Conclusions

This prospective clinical study was performed with an early loading protocol using a complete digital workflow, resulting in a high survival rate of implants in the mandibular posterior region. In addition, ISQ values, marginal bone level, and peri-implant soft tissue parameters showed acceptable results during the 1-year follow-up period. Within the limitations of this study, all three types of taper implants with an early loading protocol showed a successful treatment modality, prevailing slightly in ISQ values after loading in a novel macro-design implant (experimental groups) compared to the control group.

## Figures and Tables

**Table 1:** Demographic data of study participants.

Data	Variables	Control (Straumann, SLActive Bone Level Implant)	Experiment 1 (Neobiotech CMI IS-III Active Implant)	Experiment 2 (Neobiotech CMI IS-III HActive Implant)	*P*-Value
Subject Based (N = 24)	Participant Number	7	9	8	-
	Age (mean ± SD)	51.57 ± 14.28	46.89 ± 10.88	53.63 ± 6.72	
	20–60	5	9	6	0.437
	Over 60	2	-	2	0.795
	Sex				
	Male/Female	5/2	6/3	2/6	0.125
Implant Based (N = 24)	Implant number	7	9	8	
	Lower				
	1st premolar/2nd premolar	-	0/1	0/1	
	1st molar/2nd molar	2/5	2/6	3/4	0.829
	Implant Type	∅4.1 × 8 mm (1)	∅4.0 × 10 mm (1)	∅4.0 × 10 mm (1)	-
		∅4.8 × 8 mm (4)	∅5.0 × 8.5 mm (3)	∅5.0 × 8.5 mm (3)	
		∅4.8 × 10 mm (2)	∅5.0 × 10 mm (4)	∅5.0 × 10 mm (4)	
			∅5.0 × 11.5 mm (1)		

**Table 2:** Comparison of marginal bone loss among three different types of implants.

		Control (Straumann, SLActive Bone Level Implant)	Experiment 1 (NeobiotechCMI IS-III ActiveImplant)	Experiment 2 (NeobiotechCMI IS-III HActiveImplant)		
**Number of Participants**		**7**	**9**	**8**		
Duration	Area	Mean ± SD (mm)	Mean ± SD (mm)	Mean ± SD (mm)	*P*-value *	Pairwise ** (*P* < 0.017)
12-week follow up	Mesial	0.36 ± 0.22	0.06 ± 0.10	0.37 ± 0.42	0.015	I
	Distal	0.43 ± 0.31	0.45 ± 0.44	0.67 ± 0.40	0.558	-
	Average	0.39 ± 0.16	0.26 ± 0.24	0.52 ± 0.40	0.247	-
24-week follow up	Mesial	0.27 ± 0.23	0.09 ± 0.18	0.35 ± 0.28	0.075	-
	Distal	0.51 ± 0.29	0.47 ± 0.43	0.53 ± 0.30	0.698	-
	Average	0.39 ± 0.20	0.28 ± 0.30	0.44 ± 0.28	0.256	-
48-week follow up	Mesial	0.17 ± 0.28	0.13 ± 0.15	0.31 ± 0.26	0.376	-
	Distal	0.39 ± 0.33	0.28 ± 0.24	0.37 ± 0.24	0.515	-
	Average	0.28 ± 0.25	0.20 ± 0.15	0.34 ± 0.22	0.424	-

* *P*-values were calculated using the Kruskal–Wallis test. ** Mann–Whitney U test; I: control vs. experiment 1; according to the Bonferroni correction method (significance level: *P* < 0.017); Area: the radiographic measurement area for calculation of marginal bone loss; Avg., the average value of mesial and distal bone loss; SD, standard deviation, Kruskal–Wallis test.
